# Genetic and Epigenetic Changes in Human Prostate Cancer

**Published:** 2011-02-01

**Authors:** S Koochekpour

**Affiliations:** 1 Department of Urology and Stanley S. Scott Cancer Center, School of Medicine, Louisiana State University, Health Sciences Center, New Orleans, USA

**Keywords:** Genetics, Epigenetics, Genome, Somatic, Germline, Prostate Cancer

## Abstract

Acquired or inherited genetic alterations either alone or in combination with epigenetic alterations are associated with prostate carcinogenesis and its progression toward advance metastatic or castration-resistant disease. A major objective of translational cancer research in post-genome era is to discover the repertoire of genetic and epigenetic variations associated with prostate cancer. Genome-wide association studies have been at least partially successful in identifying potential germline polymorphisms and allelic imbalances such as microsatellite instability and loss of heterozygosity associated with prostate cancer susceptibility. Epigenetic mechanisms such as DNA hyper- or hypomethylation and histone modifications are reversible genetic alterations which allow stable inheritance of cellular phenotypes without any changes in the DNA sequence or quantity. Epigenetic modifications can potentially be used for the molecular classification, detection, and risk assessment in prostate cancer. Chemical inhibitors of DNA methyltransferases and histone deacetylases have been used in different clinical trials and hold promise as novel chemotherapeutics to be effective alone or in combination with other therapeutic interventions in prostate cancer.

## Introduction

Prostate cancer (PCa) is the most frequently diagnosed non-skin tumor and the second leading cause of cancer-related deaths in the male population in most Western countries.[1] With the increasing usage of prostate-specific antigen (PSA) testing, there is an increased tendency to diagnose PCa in developing countries.[1][2] Race, family history and age are the unequivocally accepted risk factors for PCa. It is well established that PCa does not affect racial/ethnic populations similarly. In 2007, PCa was accountable for 37% of all cancers in African-American men in the United States.[2] African American men have a 1.6-1.9 times higher incidence rate and 2-3 times greater mortality rate than Caucasians. These findings showed to be persistent for more than two decades, before and after the PSA era.[3] Black men of West African ancestry from the Caribbean and South America share a similar incidence and mortality rate when compared to African American men. In this ethnic group, PCa presents with a higher tumor volume, more advanced tumor stage, a higher Gleason score, and a higher PSA. Overall, African American men have a worse prognosis than their Caucasian counterparts. The underlying reasons for such disproportionate ethnic differences in PCa prognosis and mortality are unclear. In part, genuine racial differences in cancer genetics and biology, socio-cultural differences, and/or access to health care systems are responsible but these factors do not totally explain the higher mortality rate in African Americans with PCa.

**Fig. 1:**
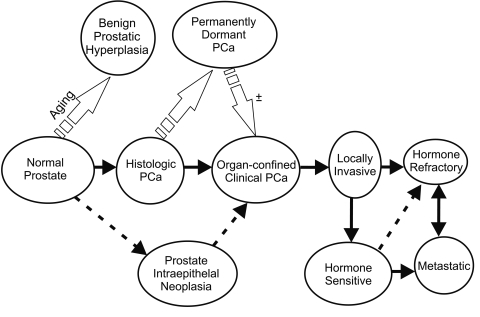
Multistep prostate cancer development and progression

PCa is known to be an indolent disease, but up to 30% of the tumors progress aggressively. PCa typically initiates as androgen-sensitive lesions but frequently develops into androgen-insensitive lesions with progression to an advanced stage (Figure 1). Androgens and other steroid hormones, acting via their receptors, regulate the development and maintenance of the differentiated functions of the male reproductive system and have been implicated in PCa development and progression.[3] Currently, available methods for PCa treatment aim to inactivate the androgen receptor (AR) by androgen deprivation or blockade with anti-androgens. The majority of the patients with early metastatic disease could be treated with androgen-deprivation therapy which leads to a significant reduction of androgen-responsive cancer cells. Androgen-deprivation via chemical or surgical castration leads an initial and clinically satisfactory treatment. However, the tumor almost always becomes hormone-refractory and more aggressive in the later stages, leading to a poor prognosis, incurable disease, and death.[4] However, contrary to localized PCa which can be effectively treated with radical prostatectomy or other modalities, hormone-refractory disease does not have effective therapeutic options. Currently, available therapeutic approaches for advanced or metastatic stages of PCa are only palliative rather than curative.

Normal prostate gland aging eventually leads to benign prostatic hyperplasia (BPH) in adult males. Malignant development of the prostate may originate from prostate intraepithelial neoplasia (PIN) which, in turn, may remain as a clinically or histologically dormant lesion, progresses into organ-confined or a locally invasive tumor, and finally, evolve into hormone-refractory or metastatic disease.

**Fig. 2:**
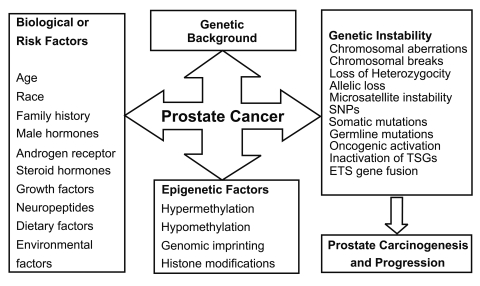
Possible risk factors in prostate carcinogenesis and progression

Failure of endocrine therapy and tumor progression is characterized by androgen-independent growth despite the presence of high levels of AR expression in metastatic disease. The persistent expression of PSA, as an androgen responsive gene despite maximal androgen blockade has led many researchers to investigate alternative signaling pathways for the AR activation in PCa. Although the role of androgen is important, it is insufficient to maintain normal prostate homeostasis by itself. This process also requires complex interactions between peptide growth factors and other growth modulators that may be regulated either by androgens or other factors. Additionally, a number of neuropeptides produced locally by neuroendocrine cells can also stimulate mitogenesis and affect the biological behavior of PCa cells.[5] As in many other cancers, genome-based technologies and approaches have demonstrated the accumulation of a variety of genetic defects, hypermutator phenotypes, and epigenetic processes (e.g., hypermethylation of tumor suppressors, hypomethylation of oncogenes, histone modification, genomic imprinting) that would eventually create the gene-specific or genome-wide genetic instability in the form of microsatellite instability (MSI), loss of heterozygosity (LOH), allelic loss (AL), single nucleotide polymorphisms (SNPs), somatic and germline mutations, chromosomal aberrations (e.g., chromosomal breaks, translocaions, recombinations), and ETS gene fusions during the natural history of PCa (Figure 2).

Natural history of prostate cancer appears as a versatile phenomenon with numerous contributing risk and/or etiological factors. These factors include age, family history, race, dietary and environmental elements, steroid hormones and their receptors, and growth regulators in connection with genetic make up and epigenetic processes which eventually lead to genetic instability and subsequently, to the progressive disease.

Indeed, PCa resembles proteus by having a varied nature, the ability to assume different forms and the ability to display of great diversity of clinicohistopathological behaviors which challenge clinicians. During the last decade, significant progress has been made in understanding the underlying biological and molecular genetic mechanisms involved in prostate carcinogenesis and its progression toward an advanced or metastatic stage. It is anticipated that by knowing the molecular signature of a PCa patient, we would be able to develop reliable predictive models for PCa prognosis, clinical behavior of the disease, and response to treatment, and to provide an individualized, targeted intervention.

In this review, I focus on current knowledge of genetic and epigenetic alterations in human PCa and discuss their potential or practical implications for PCa screening, diagnosis, and treatment.

### A. Genetic alterations in prostate cancer

PCa development results from the progressive accumulation of multiple genetic events. These events provide a hospitable soil for additional genetic alterations in the tumor microenvironment. Modern genetic and genome-based technologies have provided evidence for the presence of somatic alterations and germline variations which not only individualize PCa, but also serve as a driving force for prostate carcinogenesis and its progression toward advance incurable stages. Gene expression and microarray analyses of somatic alterations in PCa revealed the role of certain tumor suppressor genes (TSGs) and oncogenes.

Oncogenes are essentially the product of proto-oncogenes which have been subjected to genetic changes such as mutations, deletions, rearrangements, overexpression, or amplifications. Such oncogenic conversions lead to uncontrolled growth and other exaggerated phenotypes which are most commonly associated with malignant cells. For example, a single missense point mutation in the Ras gene converts it to a potent oncogene. In addition to Ras, c-Myc, c-ErbB2 (Her2/Neu), and Bcl-2 oncogenes have been implicated in PCa (Table 1).

**Table 1:** Common somatic genetic changes in prostate cancer

**Gene**	**Chr**	**Event**	**Function**
ETS agenes	7	Fusion to AR-targeted genes (TMPRSS2) c	Transcription factors, differentiation, growth
NKX3.1 f	8p21	Inactivation	Cell growth/differentiation
c-Myc	8q	Genomic amplification	Cell growth/differentiation
PTEN e	10q23	Inactivation	Cell growth/metabolism
GST-Pi d	11	Promoter methylation	Oxidative stress response
Rb g	13q	LOH h, mutation	Cell cycle regulation
P53	17p	LOH, mutation	Cell cycle regulation, apoptosis, DNA damage detection
ETS genes	21	Fusion to AR-targeted genes (TMPRSS2)	Transcription factors, cell growth /differentiation
AR^b^	X	Mutation, genomic amplification	Cell growth/differentiation

^a^ ETS: ETS family of transcription factors

^b^ AR, androgen receptor

^c^ TMPRSS2, transmembrane protease serine type 2

^d^ GST-Pi, glutathione S-transferase pie

^e^ PTEN, phosphatase and tensin

^f^ NKX3.1, NK3 homeobox 1

^g^ Rb, retinoblastoma

^h^ LOH, loss of heterozygosity

Similarly, genetic aberrations such as mutations, deletions, haplo-insufficiency, and promoter hypermethylation have the ability to inactivate or divert the TSGs from their native role as tumor suppressors. Several TSGs have been associated with prostate carcinogenesis and its androgen-independent or hormone-refractory progression. The p53 gene, known as the guardian of the normal genome, is the most commonly mutated gene in human malignancies including PCa. The p53 gene is mutated in approximately 10-20% of primary and up to 42% of advanced PCa.[6][7][8] Among other TSGs, PTEN mutations are detected in up to 27% of localized and up to 60% of metastatic tumors.[9][10][11]

In addition to the classically known oncogenes, the AR has been considered as an oncogene that is involved specifically in prostate carcinogenesis and PCa recurrence. Due to its special role, we describe its genetic alteration in more details. Functional AR is expressed at any stage of PCa including prostate intraepithelial neoplasia (PIN), primary PCa, and metastatic PCa before and after androgen-ablation therapy. Androgen-dependent and androgen-independent activation of the AR have been proposed as major events in PCa progression. The AR gene is located on the X chromosome at position Xq11-12 and spans ~90 kb containing eight exons that code for a ~2,757 bp open reading frame within a 10.6-kb mRNA. The AR is a nuclear transcription factor and a member of the steroid hormone receptor superfamily of genes. The normal growth, development, and maintenance of the prostate are dependent on androgen acting through the AR. Loss of androgen-dependence in PCa remains a key dilemma in treating this malignancy. Several AR-related mechanisms have been proposed for metastatic or androgen-independent PCa progression including: 1) AR amplification, 2) a hypersensitive AR resulting from point mutations, 3) promiscuous mutant-AR protein activated by non-androgenic ligands and 4) AR-polymorphisms changing the response to androgen (e.g., poly-CAG repeat). The functional significance of an AR mutation in PCa is represented in the LNCaP cell line where the AR gene is mutated at codon 877 (Thr to Ala).[12] Due to this mutation, the growth of LNCaP is stimulated in vitro not only by androgens, but also by non-androgenic steroids (e.g., estrogens, progesterone) and anti-androgens. The AR is among the most mutated type of the steroid receptors. So far, more than 700 mutations of the AR have been reported, most of which led to different, non-malignant clinical categories of androgen-insensitivity syndrome.[13] Overall, AR mutations in Caucasian patients are rarely found in untreated localized PCa (<2%), but are detected at a high frequency in hormone-refractory, androgen-ablated, and metastatic tumors.[13][14] The frequency of AR mutation varies greatly among different studies, up to 25% in AD tumors and up to 50% in some metastatic hormone-refractory tumors.[15] The gain of function mutations of the AR in PCa are detected in different functional domains and rarely in 5’- and 3’-UTRs of the gene. Most of these mutations are single base substitutions that directly or indirectly affect the AR function. About 49% of the mutations are located in the LBD, 37% at the NTD, and 7% at DBD. Unlike somatic mutations, germline AR mutations are rarely identified. The result of several linkage studies of hereditary PCa have not showed a linkage to the Xq11-q12 chromosomal region of AR indicating that the AR gene may not be a major PCa susceptibility gene.[16][17] However, it does not exclude the possibility that the AR as a low to moderate peneterance, predisposing gene for PCa. The existing reports on AR germline mutations in PCa are limited to Caucasian patients. The R726L mutation was reported in Finnish patients with sporadic or familial PCa.[18][19] Additional reports include two unrelated PCa patients with G2T and C214A mutations within the 5’-UTR (non-coding) region of the AR[20] and one final report showed the AR-Q798E mutation in the PCa tissue and the gDNA of a patient.[21] AR somatic and germline mutations have been recently reported in African Americans with sporadic or familial PCa.[22][23][24] While investigating AR sequence in the E006AA cell line derived from a Gleason 6 organ-confined tumor, we observed X chromosome duplication and AR gene amplification. Somatic AR mutation (599 S>G) in this cell line was proved to be as dominant-negative or loss-of-function type.[23] In addition, in author’s laboratory, a germline AR (A1675T:T559S) substitution mutation in the DNA-binding domain was discovered for the first time in three PCa-affected members of an African-American family with a history of early-onset disease.[24]

### B. Important DNA polymorphisms in prostate cancer

In two randomly selected human genomes, only 0.1% of the DNA sequence (i.e., 3 x 10(6) bases) is not identical. This variation is known as a polymorphism. The simplest form of this variation is the substitution of one single nucleotide for another, termed single-nucleotide polymorphism (SNP). SNPs are the most common type of polymorphisms and occur with a frequency of 1 in 250 base pairs in the entire genome including the promoter region, exonic sequences, intronic sequences, and other non-coding sequences. These simple DNA sequence changes may or may not change the encoded amino acids. SNPs may influence promoter activity, DNA and pre-mRNA conformation, mRNA stability, and play a direct or indirect role in phenotypic expression. Comparative studies on identical twins, fraternal twins, and siblings suggest that genetic variations in the form of SNPs is one of the factors associated with a susceptibility to many common benign and malignant diseases such as asthma, diabetes, cancer, hypertension, migraine, and human traits such as tallness, curly hair, and individuality[25][26][27][28] (Table 2). In addition, several investigators reported important polymorphic variants of androgen-regulatory genes in PCa (Table 3).

**Table 2:** SNPs with strong association with prostate cancer

**Chr b**	**SNP a Reference #**	**Alleles (-/+) d**	**OR c**	**Function**	Refs
11q13	7931342	T/G	1.21	Intergenic sequence	25 26
19q13	2735839	A/G	1.37	Androgenic effect	27 26
7q21	6465657	T/C	1.19	Membrane trafficking	26
10q11	10993994	C/T	1.38	Tumor suppression	25 26
17q12	4430796	G/A	1.22	Tumor suppression /epithelial differentiation	27 25, 26
Xp11	5945619	T/C	1.29	Apoptosis, DNA repair, stress response	28 26
10q26	4962416	T/C	1.18	Antiapoptic properties	25
6q25	934554	C/T	1.21	Drug detoxification properties	26
17q24	1859962	T/G	1.2	Intergenic sequence	27 26
3p12	2660753	C/T	1.3	Intergenic sequence	26

^a^ SNP, single nucleotide polymorphism

^b^ Chr, chromosome

^c^ OR, odds ratio related to having an additional copy of the risk allele

^d^ Alleles (-/+), the right allele is associated with increased PCa risk

**Table 3:** Important polymorphisms of androgen-regulatory genes

**Gene**	**Polymorphism**	**Normal function**	**Effect of polymorphism**
AR d	CAG or GGC	Prostate growth/ Differentiation	Structural change in AR d
	726(R>L)		Changes in transcriptional activity of AR
	1733(g>a)		Increased PCa risk
	748 (a>t)		Reduced AR stability
CYP17 e	27(t>c) (-34) 5’-UTR	Steroid metabolism	By generating one more SP1-binding motif increases transcription rate
CYP1B1 f	355(g>t)	Testosterone hydroxylation	Amino acid substitution
	100 (c>t)		Unknown
	263(g>a)		Unknown
	13(c>t)		Unknown
	142(c>g)		Amino acid substitution
CYP3A4 g	CYP3A4*1B [392(a>g)]	Oxidative metabolism of testosterone	Unknown / SNP b in the promoter
CYP3A5 h	CYP3A5*3	Metabolic detoxification or clearance	Reduction of enzymatic activity/defect in splicing
CYP3A43	CYP3A43*3 31867(c>g)	Testosterone metabolism	Amino acid substitution
SRD5A2	TA (n) repeats at 3’-UTR c	5α-reductase/converts testosterone to DHT a	Unknown

^a^ DHT, dihydrotestosterone

^b^ SNP, single nucleotide polymorphism

^c^ UTR, untranslated region of gene

^d^ AR, androgen receptor

^e^ CYP17, cytochrome P450C17α

^f^ CYP1B1, cytochrome P4501B1

^g^ CYP3A4, cytochrome P4503A4

^h^ CYP3A5, cytochrome P4503A4

A relatively long fragment of DNA with approximately 100 kilobases and a distinctive set of SNPs for any given location of a chromosome is called a haplotype. Haplotype diversity among individuals may be generated by new SNP alleles originating secondary to mutations at various loci.[29] The technological development of SNP microarrays has enabled simultaneous detection of a significantly large number of polymorphic loci. SNP genotyping methods allow investigators to detect copy number changes and signal intensity variations together with the changes in allelic composition, loss of heterozygosity (LOH), and linkage analysis of cancer susceptibility loci. While the high density resolution of SNP arrays is clearly an advantage, like other DNA array-based methods, SNP microarray analyses have several limitations including the inability to detect balanced chromosomal translocations, inversions, and whole-genome ploidy changes. Several investigators have provided evidence in support of the association between SNPs and sporadic, hereditary, or familial PCa. Other studies have been carried out to predict PCa risk with genome-wide association (GWA) SNPs. The overall magnitude of effect for individual GWA SNP is small and might only account for 15% risk; for familial PCa; therefore, due to their low value for predicting familial PCa risk, it is unlikely that they may account for high percentage of sporadic PCa in the general population. However, a combination of individual SNPs which are independently associated with PCa can significantly (2 to 4-folds) increase the risk of PCa among the carriers of risk-alleles.[30][31][32]

### Polymorphic CAG/GGC repeats of AR gene

Androgens are native ligands for the AR. The most variable region of the AR is the N-terminal domain (exon 1) with 555 amino acid residues. This domain has several regions of highly repetitive DNA sequences. Two of the most highly recognized AR gene polymorphisms are the (CAG)n and (GGC/N)n repeats. The most important is a CAG triplet that begins at codon 58 with an average of 22 repeats. In vitro studies have demonstrated an inverse relationship between the length of both repeats and the AR activity level.[33] With respect to CAG repeats, studies have suggested that 27 alleles ranging from 5 to 31 repeats could be detected in various populations. Earlier studies indicated that short CAG repeats (≤ 22) are more prevalent in African American males (75% with short alleles; median length, 18), less frequent in European-Americans (62% with short alleles; median length, 21), and least common in Asians-Americans (49% with short repeat alleles; median length, 22);[34] thus, an AR with shorter CAG repeat lengths is more prevalent in racial groups with higher PCa risk. Short CAG repeat lengths also correlate with early onset of PCa suggesting that early tumorigenesis is dependent on a more active AR.[11][12] The biological significance of polyglycine (GGC/N) repeat in the exon 1 is less clear; hence, some studies have proposed that the size of the glycine repeats might increase the PCa risk.[35][36][37] Chang et al. suggested that AR alleles with ≤ 16 GGC repeats are associated with PCa risk. However, it was most recently revealed that there is no significant association between CAG/GGC polymorphisms and PCa risk in African Americans or Caucasian Americans.[36][37][38]

### Cell-cycle related genes

SNPs of cell cycle regulating genes have been investigated in several PCa association studies. Kibel et al investigated nine SNPs of TP53, CCND1, CDKN1A, CDKN1B, CDKN2A, and MDM2 and discovered that the t allele of MDM2 was the most promising allele associated with PCa.[39] The presence of at least one copy of the t allele of MDM2 tSNP309g was associated with an increased risk of advanced PCa (odds ratio [OR] 2.26, 95% CI 1.15–4.46). This association was particularly strong in hormone-refractory PCa (OR 2.28, 95% CI 1.01–5.12) and a younger age of diagnosis. The MDM2 tSNP309g is located in the promoter region and increases the affinity for the transcription factor Sp1 which increases MDM2 expression levels. In another study analyzing six SNPs in p53, p21, MDM2, PTEN, GNAS1, and bcl2 genes, there was a significant increase in the frequency of the GNAS1 C/C genotype in PCa patients compared with control subjects.[40]

### Cell-adhesion regulatory genes

Among the cell adhesion molecules, the E-cadherin is a highly polymorphic gene. Its aberrant expression is associated with malignant prostate transformation, metastatic potential of primary PCa, and poor prognosis.[41][42][43] The -160C/A polymorphism of E-cadherin promoter has been intensively studied in carcinomas. The transcription activity of the A allele is 68% less than the C allele.[44] Verhage et al. have demonstrated that the A allele frequency was significantly higher in PCa patients than in the control subjects.[45] The A-allele carriers had a relative risk of 4.6 (2.3–9.3) for sporadic and 2.2 (0.9–5.2) for hereditary PCa.

The intercellular adhesion molecules (ICAMs) are a group of proteins involved in cell adhesion and signaling associated with several human cancers, including endocrine-regulated breast and PCa.[7][8][46] Expression alterations in these adhesion molecules may lead to the destruction of tissue architecture and distant metastasis.[48][49] Chen et al. investigated the association between ICAM genes polymorphisms and risk of PCa. They identified that two SNPs (−9 A/C and K469E) in the ICAM-1 gene that were associated with PCa risk in men who had a positive family history of PCa. In addition, there was an increased risk of disease for those with the CC genotype (-9 A/C variant) of the ICAM-1 gene and those with at least one G allele of non-synonymous K469E variant. Finally, they found a significant association in PCa for a common haplotype within the ICAM gene cluster containing the -9 A/C variant.[47]

### Angiogenesis regulatory genes

Neoplastic growth is dependent on the formation of new blood vessels (neovascularization) for oxygen and nutrient supply. Cancer cells stimulate angiogenesis, a complex multistep process that involves various angiogenic factors and matrix-degrading proteolytic enzymes.[50] Vascular endothelial growth factor (VEGF) is one of the most potent angiogenic factors. The association between various SNPs of VEGF (-1154AA and -634CG) and PCa has also been investigated. In two independent studies,[51][52] the VEGF -1154AA genotype proved to be less frequent in PCa patients than in the control subjects. In addition, patients with the -1154A-allele were significantly less susceptible to high-grade tumors when compared with non-carriers. On the contrary, a significantly increased PCa risk was associated with the VEGF-634 (GC+CC) combined genotype. The VEGF -634C allele was associated with an aggressive phenotype of PCa. Together, the VEGF haplotype (-1154A/-634G) was negatively associated with risk of PCa risk and aggressive tumors. Additional studies also reported the association between other SNPs for angiogenic factors (e.g., various isoforms of nitric oxide synthesis, epidermal growth factor) and PCa risk.[53][54]

### Vitamin D pathway-related genes

Several studies have demonstrated a chemopreventive role for Vitamin D in PCa. Vitamin D exerts its anti-tumor effect by increasing apoptotic-cell death, inhibiting cell cycle progression, interacting with the insuline-like growth factors, and decreasing the metastatic potential of PCa. The association between Vitamin D and PCa risk remains controversial.[55][56][57] The anti-proliferative effects of activated Vitamin D are thought to be mediated through a pathway that involves Vitamin D Receptors (VDRs).[58] It is possible that VDR gene polymorphisms could affect the binding of biologically active vitamin D and modulate the anti-proliferative effects of Vitamin D. Several studies focused on the six polymorphic loci (Cdx2, FokI, BsmI, ApaI, TaqI, and the poly A microsatellite) of the most common VDR variants. Initial reports revealed an association of the TaqI tt and poly (A) SS genotypes with decreased PCa risk.[59][60] In another study on the Brazilian population, there was no association between the Apal and Taql polymorphisms between PCa patients and controls.[61] In a population-based study, the frequency of the BsmI, FokI, and poly A genotypes were found to be similar in PCa patients and control subjects with no association between these variants and PCa.[62] However, further stratification showed that in men with localized PCa, the BsmI bb genotype was associated with a modest increase in risk when compared with the BB genotype.

### C. Microsatellite instability in prostate cancer

In general, loss or gain of chromosomes, could be studied by Karyotype, comparative genomic hybridization (CGH) or fluorescent in situ hybridization (FISH). However, allelic imbalances such as microsatellite instability (MSI) and loss of heterozygosity (LOH) on chromosomes can be further evaluated and located with the help of microsatellite DNA markers. Microsatellites are genetically unstable, short, polymorphic, tandem repeat DNA segments distributed throughout genome. MSI was prominently demonstrated in hereditary non-polyposis colorectal cancer (HNPCC) and has been associated with defects in certain DNA repair genes.[63][64] Conservation of genomic stability is at least partially dependent on a specific class of DNA mismatch repair (MMR) genes which are responsible for correcting the misincorporated nucleotides caused by either a replication error or any chemical induction.[65] Structural and functional defects of MMR genes lead to the loss of MMR proteins, down-regulation of MMR enzyme activity, and a lower expression of MMR-related genes. Collectively these defects are frequently associated with a higher mutation rate (100-1000 fold) and tumorigenesis.[66] Microsatellite analyses have shown frequent allelic losses on the short or long arm of different chromosomes (e.g., 1q, 3p, 5q, 6q, 7q, 8p, 10q, 13q, 16q, 17p, 17q, 18q) in PCa. Studies have indicated that the average allelic losses on these chromosomes range from approximately 13% for 17q to 36% for 8p in primary tumors and 27% for 17q to 61% for 13q in metastatic tumors. Overall, the highest allelic loss was 42% and 40% at 16q and 8p regions, respectively. Since the allelic imbalance on these two loci has also been associated with PCa linkage studies, it suggests the presence of TSGs.

### D. ETS gene fusions

Oncogenic translocations, the abnormal fusion of two genes, result in the formation of chimeric protein with dysregulated gene expression and a high potential for transforming abilities. In most cases the involved genes are transcription factors that bind to their specific target DNA sequences, mostly located in the promoter regions of genes and increase or decrease their expression. The ETS transcription factors can change the expression of several proteins which are involved in a variety of signaling pathways regulating cell growth, senescence, apoptosis, motility, and carcinogenesis. Following gene fusion, the ETS transcription factors abnormally activate or silence their specific target genes leading to oncogenic transformation.[67] The discovery of ETS gene fusions has improved our understanding of early PCa development. Fusion of the AR-regulated gene transmembrane protease, serine 2 (TMPRSS2) to the ETS family member, ERG (v-ets erythroblastosis virus E26 oncogene homolog) is observed in approximately half of PCa. The TMPRSS2-ERG fusion results in a high expression of the ERG gene and is believed to serve as a driving force behind prostate tumorigenesis.[68] Other groups have also identified such translocations in a high percentage of PCa patients.[69][70] Additional reports support the presence of ETS translocations in aggressive tumors and associates with high PCa-specific mortality.[69][71][72] The fusion events that involve ETS family members are likely to be important during early carcinogenesis events and continue to exist in metastatic and castration-resistant PCa. So far, no universally consistent association exists between ETS fusions and the clinical outcome of the disease. It appears that in addition to the ERG gene status, other factors or variables play major roles for poor clinical outcome. Analysis of the TMPRSS2-ERG expression signature in PCa appears to involve β-estradiol signaling and histone deacetylation.[73][74] The involvement of epigenetic processes in the modulation of TMPRSS2-ERG activity remains to be investigated.

### F. Epigenetic changes in prostate cancer

Epigenetic refers to mechanisms that allow the stable transmission of cellular traits without a change in the DNA sequence or quantity. Overall, epigenetic defects reported in cancers include a) reactivation of embryonic genes, b) loss of imprinted genes changing inactive and active alleles, c) dysregulated expression of micro-RNAs, d) increased gene recombination, and e) transcriptional silencing of TSGs and housekeeping genes. Epigenetics encompasses several different phenomena, such as DNA methylation, histone modifications, RNA interference, and genomic imprinting. Epigenetic processes regulate gene expression and can change malignancy-associated phenotypes such as growth, migration, invasion, or angiogenesis. Epigenetic events may also play a role in benign prostatic hyperplasia (BPH). Epigenetic regulatory mechanisms appear very sensitive to external stimuli or influences such as diet and oxidative stress.

### DNA methylation status and prostate cancer

**Fig. 3:**
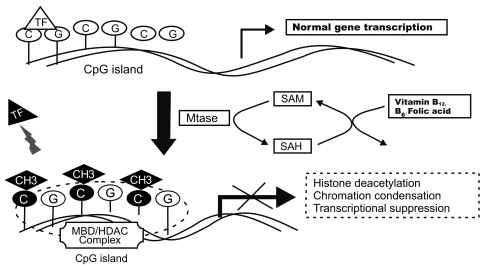
DNA methylation and cancer

DNA methylation is the addition of a methyl (-CH3) group to the 5’-carbon of cytosine, adjacent to guanine in CpG sequences, and catalyzed by a series of conserved enzymes known as DNA methyltransferases (MTases). Most CpG islands are unmethylated in eukaryotic cells. The CpG island methylation occurs in about 1% of the genome, which is less than the expected statistical fraction (i.e., 6%). Methylcytosine residues are often found in short stretches of CpG-rich regions (i.e., CpG islands) that are 0.5-2 kb long and in the 5’ region of approximately 60% of genes. The CpG dinucleotides, present in the genome, are commonly clustered in groups or CpG islands located mostly at or near the promoters (~60%) and initial exons of many genes. DNA methylation changes can occur as either hypo- or hypermethylation; however, both forms can lead to genetic instability and transcriptional gene silencing and both have been implicated in a variety of human cancers, including PCa.[75] The methyl group is supplied by S-adenosylmethionine (SAM) (Figure 3) which then is recycled via a folate- and cobalamin-dependent pathway. Hypomethylation or loss of methylation can be accelerated by altering this regenerative process through dietary deficiencies of folic acid, Vitamin B12, or other substrates. During this process, DNA methylation acts as a stable tag on the promoter of a gene and recruits methyl-binding proteins (MBD) and other proteins, such as histone deacetylases (HDACs) to form large-scale heterochromatic structures that silence the associated genes.

The clustered CpG site (CpG island) and transcription start site (arrow) of a hypothetical active gene is shown. Methylation occurs on 5’-carbon of cytosine adjacent to guanine. DNA-methyl transferase (MTase) methylates the CpG island which recruits the methyl binding domain (MBD) and histone deacetylases (HDACs) to the methylated DNA and leads to histone deacetylation, condensation of chromatin, loss of transcription factor (TF) binding, and silencing of the gene expression in cancer and other premalignant conditions. MTase converts S-adenosylmethionine (SAM) to S-adenosylhomocysteine (SAH). A dietary supply of vitamins B12, B6, and folic acid via several steps regenerate SAH to SAM.

Hypomethylation predisposes cells to chromosomal aberrations, such as chromosomal breaks, translocations, and aneuploidy (Figure 2). Methylated silencing of proto-oncogenes or other cancer promoting genes (e.g., H-RAS) could be reversible and lead to the activation of such genes.[76] Traditionally DNA MTases are divided into two categories, de novo enzymes which are responsible for establishing new methylation patterns and maintenance enzymes which essentially replicate the existing patterns.[77] DNA MTase 1 (DNMT1) is a maintenance enzyme, and DNA MTase 3a and 3b (DNMT3a and DNMT3b) perform de novo functions. The DNMT1 activity is increased up to 3-folds higher in PCa tissues and cell lines than in BPH tissues and cell lines.[78] DNMT2 and DNMT3a are increased in the LnCaP-r (hormone refractory PCa) than in the androgen-sensitive parent cells (LNCaP) which suggests that changes in the methylation status may be involved in the androgen-independent progression of PCa. A recent report showed that polymorphism in the DNMT3b increased the risk of PCa development by 2.8 folds. Table 4 lists several important genes that are either silenced or inactivated by hypermethylation in PCa. Interestingly, many of the hypermethylated genes are also mutated or deleted in PCa.[79][80][81][82]

Some reports have indicated that methylation changes may be more important in prostate carcinogenesis rather than its metastatic progression.[83] Hypermethylation in the high grade prostatic intraepithelial neoplasia (HPIN) has been found at GSTpi,[84] an important gene that is involved in the detoxification of electrophilic compounds, such as carcinogens and cytotoxic drugs, by glutathione conjugation. Hypermethylation of GSTpi is one of the most frequent epigenetic alteration, with a frequency of 70% to 100% in clinical PCa specimens.[83][85] Progressive accumulation of methylated alleles from high grade prostatic intraepithelial neoplasia to advance PCa occurs in 14-3-3σ gene, suggesting a role for this epigenetic event in prostate carcinogenesis.[79][80][81][82][86] (Table 4) In addition to these, more than forty other genes have been reported as targets of hypermethylation silencing in PCa.[87]

**Table 4:** Hypermethylated genes in prostate cancer

**Gene**	**Chromosome**	**Role/Function**	**Hypermethyaltion**	**Refs**
14-3-3σ	1p36.11	Cell cycle	99%	76
GSTM1 a	1p13.3	Glutathione-S-transferase	58%	98
RASSF1α c	3p21.3	Tumor suppressor	Up to 79%	83
RARβ d	3p24	Nuclear hormone receptor	66%	76
APC j	5q21-q22	Tumor suppressor	100%	79
ERα h	6q25.1	Estrogen receptor	95%	80
MDR1 e	7q21.1	ABC-transporter	Up to 100%	79
GSTpi b	11q13	Glutathione-S-transferase	Up to 100%	83 79
CD44^f^	11p13	Cell adhesion	72%	81
hSPRY2 g	13q31.1	Inhibitor of cell growth	Up to 82%	82
ERβ i	14q23.2	Estrogen receptor	up to 100%	80
E-cadherin	16q22.1	Tumor suppressor	Up to 72%	81

^a^ GSTM1, Glutathione S-transferase

^b^ GSTpi, Glutathione S-transferase P1 (pi)

^c^ RSSF1α, Ras association domain family 1 gene

^d^ RARβ, Retinoic acid receptor beta gene

^e^ MDR1, multi-drug resistance 1

^f^ CD44, cluster differentiation 44

^g^ hSPRY2, human sprouty homolog 2

^h^ ERα, estrogen receptor alpha

^i^ ERβ, estrogen receptor beta

^j^ APC, adenomatous polyposis coli

### Histone Modifications in Prostate Cancer

Another distinct epigenetic change that is linked to DNA methylation is the modified state of the surrounding histones in which the DNA is packaged. The N-terminal tails of these highly conserved histone proteins could be covalently or reversibly modified by the addition or removal of acetyl, methyl, ubiquitin and adenosine diphosphate-ribosyl groups. This unique complex modification pattern (histone code) exists in balance and functions to regulate transcriptional activity levels of the genes.[88] The addition of acetyl groups to the N-terminal lysines of the histones creates an open chromatin conformation which leads to a transcriptional activation of the genes. Additionally, histone hypoacetylation is associated with DNA methylation. Primarily the addition of acetyl group on histones H2a, H3 and H4 is catalyzed by enzyme histone acetyl transferases (HATs). The HATs p300, PCAF, and Tip60 up-regulate AR expression or activity which leads to androgen-independent signaling of AR.[88][89]

HDACs, which are frequently upregulated in PCa, remove the acetyl groups from lysines on the histones.[90] The expression levels of HDAC mRNA and proteins are increased up to 3-folds in PCa as compared with BPH tissues and cell lines.[91] The highest levels of HDAC1 are detected in hormone refractory PCa which supports a role for this epigenetic modification in androgen-independent PCa.[92] Histone methylation on a specific location of the histone tail is another type of histone modification. Additionally, the expression of histone methyltransferases is changed in PCa. EZH2 is a prototypical example of histone methyltransferase that methylates lysine 27 on histone H3.[93] Methylation of this lysine is a marker of increased gene expression. Additionally, EZH2 expression is up-regulated in metastatic hormone-refractory PCa and its increased expression in primary cancers predicts biochemical recurrence.[94]

### Genomic Imprinting in Prostate Cancer

Imprinting is a normal cellular process that is based on the maternal or paternal DNA of origin; one allele is silenced and the other one is expressed. There are more than forty known human genes that demonstrate genomic imprinting. The IGF2 and p57 genes are imprinted in many human tissues and it has been reported that IGF2 presents with two alleles in PCa as compared with the normal single allele imprinted pattern in BPH.[95] Therefore IGF2 imprinting may increase the chance of prostate carcinogenesis. Loss of IGF2 imprinting also occurs with the aging of the prostate and during senescence in vitro.[96] Inhibitors of DNA MTase can reverse the loss of imprinting and modulate IGF2 expression. The p57 tumor suppressor gene expression is frequently changed in PCa and its gene imprinting is regulated by DNA methylation.[97]

### G. Diagnostic and prognostic aspects of epigenetic alterations in prostate cancer: Epigenetic Diagnostic Markers

PSA is the best available marker, but it cannot effectively differentiate between PCa and other benign conditions such as BPH and prostatitis. Therefore the false positive PSA test can lead to expensive and invasive approaches such as transrectal prostate biopsy. Among other genetic alterations, aberrant DNA methylation potentially might have the potential as diagnostic epigenetic marker for PCa and could be tested in tumor tissues and body fluids (e.g., serum, urine). The methylation markers have several advantages when compared with those genetic markers based on sequence changes or variants (e.g., mutation, SNPs, etc). The mthylation markers are simple in nature; with high sensitivity, these markers can be detected, either quantitatively or qualitatively, by available well-established techniques (e.g., PCR). Aberrant DNA methylations are more frequent than mutations and can be identified by genome wide screening methodologies. The GSTpi gene is a widely investigated tumor marker widely. GSTpi is PCa-specific, hypermethylated, and silenced in more than 90% of PCa patients.[98] GSTpi methylation is also detected in the proliferative inflammatory atrophy of the prostate, which is a known linked to prostate carcinogenesis.[99] The GSTP1 hypermethylation is reported in the serum samples of 72% of PCa patients, 78% of patients with locally invasive or advanced PCa, and following prostatic massage, in the urine samples of 68% of patients with early stage disease.[99] In addition to GSTPI, the methylation status of RARβ, CD44, E-cadherin (ECAD), RASSF1A, APC, and the tazarotene induced gene 1 (T1G1) have been investigated in PCa patients. Other studies have demonstrated that a combination of methylation markers in body fluids and tissues, including GSTpi improves PCa diagnosis. It has been reported that a combination of the methyaltion analyses of four genes (MGMT, p16, ARF, and GSTP1) in the urine could detect 87% of PCa cases with 100% specificity.[100] Additional detailed investigations are needed to systematically identify methylation markers for routine clinical and histopathological diagnosis.

### Epigenetic Prognostic Markers

In addition to the potential diagnostic value, GSTpi hypermethylation is detected in 40% of pre-operative bone marrow aspirates in patients with advanced PCa and in 90% of PC patients with lymph node involvement.[95] Moreover the methylation status of CD44, CAV1, T1G1, and CDH1 may also be useful in the molecular staging and prediction of disease progression.[101] High grade PCa cases are associated with the hypermethylation of several genes, including RASSF1A, GSTP1, RARβ, and CDH13.[102] A recent report discovered that GSTpi hypermethylation was a significant predictor of PSA recurrence in PCa patients.[95] Multivariate analyses of APC or GSTpi hypermethylation in 74 PCa patients significantly predicted the time needed for disease progression following radical prostatectomy. Bastian et al., in a study using matched tumor and normal tissues of 53 patients, discovered that the combined hypermethylation of GSTpi, APC, and PTGS2 gene correlated with other clinical or pathological variables (e., pT-stage, Gleason score), with 71.1% to 96.2% sensitivity and 92.9% to 100% specificity.[103] APC and PSTG2 methylation status appear to have a higher prognostic value of PCa in several studies completed to date. In addition to these promising hypermethyalted genes, global histone modifications may be associated with the risk of PCa recurrence.[104] For example; increased global acetylation and histone methylation on histones H3 and H4 permit the stratification of patients with low grade tumors into different risk groups for recurrence. Interestingly, these patterns of histone modification were predictors of outcome independent of stage, preoperative-PSA, and capsular invasion.[105] The predictive value of simulataneously several methylation makers have been shown in other studies.[106]

### H. Therapeutic targeting of epigenetic processes for prostate cancer prevention and treatment

Unlike irreversible genetic changes such as mutations, epigenetic changes are reversible and could be approached therapeutically. Theoretically, reversing these changes allows for the reactivation of TSGs or silencing oncogenes. Most chromatin modifying drugs have been studied in hematological malignancies.[106] In PCa, several in vitro and in vivo studies have used pharmacological agents to reverse epigenetic processes. Table 5 lists inhibitors of DNA MTase enzymes and HDACs that are used in phase I or II clinical trials. Nucleoside analog inhibitors of DNA MTase, such as 5-Azacytidine (5-AC) and 5-aza-deoxycytidine (decitabine or DAC) have been extensively used in vitro model systems to reverse abnormal hypermethylation and restore the expression of silenced genes; however, clinical success was limited with these drugs (Table 5).[107][108][109][110][111][112][113][114] In a phase II study, 14 patients with recurrent metastatic PCa, following complete androgen blockade and flutamide withdrawal, were treated with decitabine; only two patients responded with stable disease and delayed progression time.[108] This report concluded that decitabine is a well-tolerated drug with modest clinical activity against hormone-refractory disease. Zebularine is another inhibitor of DNA MTase which is less toxic and more stable than 5-AC. Additionally, zebularine has also antiproliferative activity that will be more effective in cancer cells than in normal cells.[109][110][111][112][113][114][115] The oligodeoxynucleotide MG98, is another MTase inhibitor which binds with the 3’-UTR region of the DNMT1 mRNA and leads to its degradation before translation.[116] One disadvantage of the MTase inhibitor is that the chromosomal site of demethylation cannot be controlled. In addition, non-specificity of the hypomethylating effects of MTase inhibitors may promote cancer in some situations which led investigators to consider other possibilities, such as inhibiting specific methyl-binding proteins which recruit histone modifying enzymes to methylated regions.[104][109][110][111]

**Table 5:** Pharmacological agents modifying epigenetic processes in Phase-I and –II clinical trials

	**Clinical Trial**	**Tumor Type**	**Clinical or Biomarker Response**	Refs
DNA MTase ainhibitor				
5AC e	Phase-I	Solid tumors	No response/Decreased DNMT activity	111
MG98	Phase-I /-II	Solid tumors	Demethylation/ Disease stabilization in 2/23 patients	112
DAC f	Phase-II	HRPCa d	Disease stabilization in 2/14 patients	113 114
HDAC b inhibitors				
PB g	Phase-I	Prostate cancer Solid tumors	Disease stabilization in solid tumors, PSA rise in 17/19 patients	115
SAHA c	Phase-I	Solid tumors	Tumor regression (1 complete , 5 partial response)	116
MS-275	Phase-I	Prostate, lymphoma, and other solid tumors	Partial response Biomarker response	117

^a^ DNA MTase, DNA methyltransferase

^b^ HDAC, histone-deacetylase

^c^ SAHA, Suberoylanilide hydroxamic acid

^d^ HRPCa, hormone-refractory prostate cancer

^e^ 5-AC, 5-Aza-cytidine

^f^ DAC,5-aza-2’-Deoxycytidine

^g^ PB, Sodium phenylbutyrate

Increased HDAC activity in PCa provides another therapeutic opportunity. In vitro studies have demonstrated the pro-apoptotic and anti-anti-angiogenic effects of HDAC inhibitors (HDACis) such as MS-275, suberoylanilide hydroxamic acid (SAHA), and sodium phenylbutyrate (PB) (Table 5).[112][113][114] SAHA is a class I HDACi, which significantly suppresses the growth of androgen-sensitive LNCaP xenografts in nude mice[117] and PB has a pro-apoptotic activity in PCa cell lines.[118] Due to the relative tumor selectivity and less toxicity, HDAC inhibitors have been used in phase I trials in patients with advanced PCa to evaluate dose tolerance and also biomarker-response (Table 5). In a small number of patients, partial treatment response was observed in terms of tumor regression, stable disease, and biomarker response.

### Regulation of epigenetic processes by environmental and dietary factors

Environmental and dietary factors also regulate epigenetic processes. Global and gene-specific DNA methylation could be affected by carcinogenic compounds found in cigarette smoke, alcohol (ethanol), and metals such as nickel, cadmium, and zinc. Dietary supplements can change DNA methylation by regulating the pathways that produce SAM substrate (Figure 2 and 3). In vitro and in vivo studies have demonstrated that supplemental folate, choline, or methionine can affect DNA methylation.[119] Diet can abnormally change the epihaplotype during the life of an organism. Additionally, dietary supplement of Vitamins A, B6, or B12 were shown to decrease PCa development potentially by changing the SAM biosynthesis pathway or by acting as an antioxidants.[120] Overall dietary constituents such as green tea and selenium are believed to be chemopreventive and function as an antioxidant to prevent hypomethylation in cell lines, and thereby, guard the genome.[121][122]

### I. Epigenetic alterations in benign prostatic hyperplasia

Available knowledge on epigenetic changes in NPH is limited. To date, several hypermethylated genes, including MDR1, RASSF1a and 14-3-3σ, have been identified in BPH when compared to normal tissues, which suggests a role for these methylation changes in this growth dysfunction (Table 6). Global hypermethylation are significantly higher in normal prostatic epithelium than in BPH tissues.[123] So far, there is no knowledge on chromatin modifying drugs in BPH; however, the expression of HDAC1 and DNMT1 proteins in PCa is higher than in BPH. So far, it is not clear whether histone acetylation or methylation is associated with the field effect. Interestingly, hypermethylation of GSTpi and RARβ2 was discovered in a subset of histologically normal-looking stroma from radical prostatectomy specimens, supporting a role for stromal methylation in PCa progression.[79][80][81][124][125]

**Table 6:** Hypermethylated genes in benign prostatic hyperplasia

Gene	Chromosome	Role/Function	Hypermethyaltion	Refs
14-3-3σ	1p36.11	Cell cycle	100%	128
RASSF1α a	3p21.3	Tumor suppressor	29%	79
MDR1 b	7q21.1	ABC-transporter	71%	79
CD44 c	11p13	Cell adhesion	38%	81
MT1G d	16q13	Heavy metal binding	10%	81

^a^ RSSF1α, Ras association domain family 1 gene

^b^ MDR1, multi-drug resistance 1

^c^ CD44, cluster differentiation 44

^d^ MT1G, metallothionein 1G

### J. Genome-wide association studies (GWAS) and prostate cancer

Interest in genome-wide association studies (GWAS) was initiated with the observations that association studies could provide significantly greater power than linkage analyses when detecting genetic variants with minor to moderate phenotypic effects.[3] Although PCa is one of the most heritable cancers, identification of its underlying genetic causes has proved difficult. In the last several years, several GWA studies have detected a few germline SNPs that are individually associated with a relatively moderate increase in PCa risk.[26][27][28][126][127][128][129][130] If combined, these SNPs might be more informative in detecting higher risk among carriers. Moreover, expression array analyses identified many genetic alterations such as somatic mutations, chromosomal aberrations, and recurrent gene fusions.

### K. Conclusions and future directions

The linkage between epigenetic changes and prostate carcinogenesis or progression has led to new insights into PCa phenotypes and many novel molecular biomarkers that might change clinical diagnosis and management. Genetic changes are generally permanent and irreversible. Epigenetic changes are an early event in carcinogenesis which can be used to assess the risk of developing PCa, and specifically, are susceptible to changes resulting from environmental stimuli or factors such as diet and life style. The identification of epigenetic changes will eventually help us to understand better the complexity of regulatory pathways for gene expression in normal and PCa cells. Alterations in DNA methylation occur commonly in genetically identical tumors, having different phenotypes. New techniques, such as genomic fingerprinting, have the potential to identify and classify methylation changes in individual tumors. Chromatin immunoprecipitation (ChIP) microarray, so called ChIP-on-a-chip, could identify global changes in histone modifications (e.g., acetylation, methylation) and the histone code in PCa and BPH. High throughput assays such as genome-wide association studies (GWAS) will lead to the development of PCa epigenetic signatures. Epigenetic changes could be reversed by using modifiers of epigenetic processes such as DNA MTases and HDACs. Since epigenetic changes are early events in the PCa development, these modifiers (drugs) have the potential to be used in disease prevention. Ultimately, rational strategies for discovering efficient combinatorial therapies, using epigenetic modifiers along with other drugs, will ultimately lead to improvements in PCa outcomes. Such technological advances are likely to identify epigenetic changes in tumor specimens and assist urologist-oncologists in their clinical management of PCa.

## References

[1] Schröder FH (2010). Prostate cancer around the world.An overview. Urol Oncol.

[2] Jemal A, Center MM, DeSantis C, Ward EM (2010). Global patterns of cancer incidence and mortality rates and trends. Cancer Epidemiol Biomarkers Prev.

[3] Risch N, Merikangas K (1996). The future of genetic studies of complex human diseases. Science.

[4] Kruglyak L (1999). Prospects for whole-genome linkage disequilibrium mapping of common disease genes. Nat Genet.

[5] Collins FS, Brooks LD, Chakravarti AA (1998). DNA polymorphism discovery resource for research on human genetic variation. Genome Res.

[6] Marks PA, Richon VM, Rifkind RA (2000). Histone deacetylase inhibitors: inducers of differentiation or apoptosis of transformed cells. J Natl Cancer Inst.

[7] Gurova KV, Roklin OW, Krivokrysenko VI, Chumakov PM, Cohen MB, Feinstein E, Gudkov AV (2002). Expression of prostate specific antigen (PSA) is negatively regulated by p53. Oncogene.

[8] Li LC, Carroll PR, Dahiya R (2005). Epigenetic changes in prostate cancer: implication for diagnosis and treatment. J Natl Cancer Inst.

[9] Collins N, Poot RA, Kukimoto I, Garcia-Jimenez C, Dellaire G, Varga-Weisz PD (2002). An ACF1-ISWI chromatin-remodeling complex is requried for DNA replication through heterochromatin. Nat Genet.

[10] Deuring R, Fanti L, Armstrong JA, Sarte M, Papoulas O, Prestel M, Daubresse G, Verardo M, Moseley SL, Berloco M, Tsukiyama T, Wu C, Pimpinelli S, Tamkun JW (2000). The ISWI chromatin-remodeling protein is required for gene expression and the maintenance of higher order chromatin structure in vivo. Mol Cell.

[11] Havas K, Whitehouse I, Owen-Hughes T (2001). ATP-dependent chromatin remodeling activities. Cell Mol Life Sci.

[12] Veldscholte J, Ris-Stalpers C, Kuiper GG, Jenster G, Berrevoets C, Claassen E, van Rooij HC, Trapman J, Brinkmann AO, Mulder E (1990). A mutation in the ligand binding domain of the androgen receptor of human LNCaP cells affects steroid binding characteristics and response to anti-androgens. Biochem Biophys Res Commun.

[13] Gottlieb B, Beitel LK, Wu JH, Trifiro M (2004). The androgen receptor gene mutations database (ARDB): 2004 update. Hum Mutat.

[14] Bubendorf M, Kononen J, Koivisto P, Schraml P, Moch H, Gasser TC, Willi N, Mihatsch MJ, Sauter G, Kallioniemi OP (1999). Survey of gene amplifications during prostate cancer progression by high-throughput fluorescence in situ hybridization on tissue microarrays. Cancer Res.

[15] Linja MJ, Visakorpi T (2004). Alterations of androgen receptor in prostate cancer. J Steroid Biochem Mol Biol.

[16] Karayi MK, Neal DE, Markham AF (2000). Current status of linkage studies in hereditary prostate cancer. BJU Int.

[17] Xu J, Meyers D, Freije D, Isaacs S, Wiley K, Nusskern D, Ewing C, Wilkens E, Bujnovszky P, Bova GS, Walsh P, Isaacs W, Schleutker J, Matikainen M, Tammela T, Visakorpi T, Kallioniemi OP, Berry R, Schaid D, French A, McDonnell S, Schroeder J, Blute M, Thibodeau S, Grönberg H, Emanuelsson M, Damber JE, Bergh A, Jonsson BA, Smith J, Bailey-Wilson J, Carpten J, Stephan D, Gillanders E, Amundson I, Kainu T, Freas-Lutz D, Baffoe-Bonnie A, Van Aucken A, Sood R, Collins F, Brownstein M, Trent J (1998). Evidence for a prostate cancer susceptibility locus on the X chromosome. Nat Genet.

[18] Mononen N, Syrjäkoski K, Matikainen M, Tammela TL, Schleutker J, Kallioniemi OP, Trapman J, Koivisto PA (2000). Two percent of Finnish prostate cancer patients have a germ-line mutation in the hormone-binding domain of the androgen receptor gene. Cancer Res.

[19] Gruber SB, Chen H, Tomsho LP, Lee N, Perrone EE, Cooney KA (2003). R726L androgen receptor mutation is uncommon in prostate cancer families in the United States. Prostate.

[20] Crocitto LE, Henderson BE, Coetzee GA (1997). Identification of two germline point mutations in the 5'UTR of the androgen receptor gene in men with prostate cancer. J Urol.

[21] Evans BA, Harper ME, Daniells CE, Watts CE, Matenhelia S, Green J, Griffiths K (1996). Low incidence of androgen receptor gene mutations in human prostatic tumors using single strand conformation polymorphism analysis. Prostate.

[22] Koochekpour S (2010). Androgen receptor signaling and mutations in prostate cancer. Asian J Androl.

[23] D'Antonio JM, Vander Griend DJ, Antony L, Ndikuyeze G, Dalrymple SL, Koochekpour S, Isaacs JT (2010). Loss of androgen receptor-dependent growth suppression by prostate cancer cells can occur independently from acquiring oncogenic addiction to androgen receptor signaling. PLoS One.

[24] Hu SY, Liu T, Liu ZZ, Ledet E, Velasco-Gonzalez C, Mandal DM, Koochekpour S (2010). Identification of a novel germline missense mutation of the androgen receptor in African American men with familial prostate cancer. Asian J Androl.

[25] Thomas G, Jacobs KB, Yeager M, Kraft P, Wacholder S, Orr N, Yu K, Chatterjee N, Welch R, Hutchinson A, Crenshaw A, Cancel-Tassin G, Staats BJ, Wang Z, Gonzalez-Bosquet J, Fang J, Deng X, Berndt SI, Calle EE, Feigelson HS, Thun MJ, Rodriguez C, Albanes D, Virtamo J, Weinstein S, Schumacher FR, Giovannucci E, Willett WC, Cussenot O, Valeri A, Andriole GL, Crawford ED, Tucker M, Gerhard DS, Fraumeni JF Jr, Hoover R, Hayes RB, Hunter DJ, Chanock SJ (2008). Multiple loci identified in a genome-wide association study of prostate cancer. Nat Genet.

[26] Eeles RA, Kote-Jarai Z, Giles GG, Olama AA, Guy M, Jugurnauth SK, Mulholland S, Leongamornlert DA, Edwards SM, Morrison J, Field HI, Southey MC, Severi G, Donovan JL, Hamdy FC, Dearnaley DP, Muir KR, Smith C, Bagnato M, Ardern-Jones AT, Hall AL, O'Brien LT, Gehr-Swain BN, Wilkinson RA, Cox A, Lewis S, Brown PM, Jhavar SG, Tymrakiewicz M, Lophatananon A, Bryant SL, Horwich A, Huddart RA, Khoo VS, Parker CC, Woodhouse CJ, Thompson A, Christmas T, Ogden C, Fisher C, Jamieson C, Cooper CS, English DR, Hopper JL, Neal DE, Easton DF (2008). Multiple newly identified loci associated with prostate cancer susceptibility. Nat Genet.

[27] Yeager M, Orr N, Hayes RB, Jacobs KB, Kraft P, Wacholder S, Minichiello MJ, Fearnhead P, Yu K, Chatterjee N, Wang Z, Welch R, Staats BJ, Calle EE, Feigelson HS, Thun MJ, Rodriguez C, Albanes D, Virtamo J, Weinstein S, Schumacher FR, Giovannucci E, Willett WC, Cancel-Tassin G, Cussenot O, Valeri A, Andriole GL, Gelmann EP, Tucker M, Gerhard DS, Fraumeni JF Jr, Hoover R, Hunter DJ, Chanock SJ, Thomas G (2007). Genome-wide association study of prostate cancer identifies a second risk locus at 8q24. Nat Genet.

[28] Gudmundsson J, Sulem P, Rafnar T, Bergthorsson JT, Manolescu A, Gudbjartsson D, Agnarsson BA, Sigurdsson A, Benediktsdottir KR, Blondal T, Jakobsdottir M, Stacey SN, Kostic J, Kristinsson KT, Birgisdottir B, Ghosh S, Magnusdottir DN, Thorlacius S, Thorleifsson G, Zheng SL, Sun J, Chang BL, Elmore JB, Breyer JP, McReynolds KM, Bradley KM, Yaspan BL, Wiklund F, Stattin P, Lindström S, Adami HO, McDonnell SK, Schaid DJ, Cunningham JM, Wang L, Cerhan JR, St Sauver JL, Isaacs SD, Wiley KE, Partin AW, Walsh PC, Polo S, Ruiz-Echarri M, Navarrete S, Fuertes F, Saez B, Godino J, Weijerman PC, Swinkels DW, Aben KK, Witjes JA, Suarez BK, Helfand BT, Frigge ML, Kristjansson K, Ober C, Jonsson E, Einarsson GV, Xu J, Gronberg H, Smith JR, Thibodeau SN, Isaacs WB, Catalona WJ, Mayordomo JI, Kiemeney LA, Barkardottir RB, Gulcher JR, Thorsteinsdottir U, Kong A, Stefansson K (2008). Common sequence variants on 2p15 and Xp11.22 confer susceptibility to prostate cancer. Nat Genet.

[29] Shastry BS (2002). SNP alleles in human disease and evolution. J Hum Genet.

[30] Thomas G, Jacobs KB, Yeager M, Kraft P, Wacholder S, Orr N, Yu K, Chatterjee N, Welch R, Hutchinson A, Crenshaw A, Cancel-Tassin G, Staats BJ, Wang Z, Gonzalez-Bosquet J, Fang J, Deng X, Berndt SI, Calle EE, Feigelson HS, Thun MJ, Rodriguez C, Albanes D, Virtamo J, Weinstein S, Schumacher FR, Giovannucci E, Willett WC, Cussenot O, Valeri A, Andriole GL, Crawford ED, Tucker M, Gerhard DS, Fraumeni JF Jr, Hoover R, Hayes RB, Hunter DJ, Chanock SJ (2008). Multiple loci identified in a genome-wide association study of prostate cancer. Nat Genet.

[31] Kote-Jarai Z, Easton DF, Stanford JL, Ostrander EA, Schleutker J, Ingles SA, Schaid D, Thibodeau S, Dörk T, Neal D, Donovan J, Hamdy F, Cox A, Maier C, Vogel W, Guy M, Muir K, Lophatananon A, Kedda MA, Spurdle A, Steginga S, John EM, Giles G, Hopper J, Chappuis PO, Hutter P, Foulkes WD, Hamel N, Salinas CA, Koopmeiners JS, Karyadi DM, Johanneson B, Wahlfors T, Tammela TL, Stern MC, Corral R, McDonnell SK, Schürmann P, Meyer A, Kuefer R, Leongamornlert DA, Tymrakiewicz M, Liu JF, O'Mara T, Gardiner RA, Aitken J, Joshi AD, Severi G, English DR, Southey M, Edwards SM, Al Olama AA, Eeles RA (2008). Multiple novel prostate cancer predisposition loci confirmed by an international study: the PRACTICAL Consortium. Cancer Epidemiol Biomarkers Prev.

[32] Zheng SL, Sun J, Wiklund F, Smith S, Stattin P, Li G, Adami HO, Hsu FC, Zhu Y, Bälter K, Kader AK, Turner AR, Liu W, Bleecker ER, Meyers DA, Duggan D, Carpten JD, Chang BL, Isaacs WB, Xu J, Grönberg H (2008). Cumulative association of five genetic variants with prostate cancer. N Engl J Med.

[33] Rajender S, Singh L, Thangaraj K (2007). Phenotypic heterogeneity of mutations in androgen receptor gene. Asian J Androl.

[34] Hardy DO, Scher HI, Bogenreider T, Sabbatini P, Zhang ZF, Nanus DM, Catterall JF (1996). J Clin Endocrinol Metab.

[35] Jenster G, van der Korput HA, Trapman J, Brinkmann AO (1995). Identification of two transcription activation units in the N-terminal domain of the human androgen receptor. J Biol Chem.

[36] Chen C, Lamharzi N, Weiss NS, Etzioni R, Dightman DA, Barnett M, DiTommaso D, Goodman G (2002). Androgen receptor polymorphisms and the incidence of prostate cancer. Cancer Epidemiol Biomarkers Prev.

[37] Platz EA, Giovannucci E, Dahl DM, Krithivas K, Hennekens CH, Brown M, Stampfer MJ, Kantoff PW (1988). The androgen receptor gene GGN microsatellite and prostate cancer risk. Cancer Epidemiol Biomarkers Prev.

[38] Lange EM, Sarma AV, Ray A, Wang Y, Ho LA, Anderson SA, Cunningham JM, Cooney KA (2008). The androgen receptor CAG and GGN repeat polymorphisms and prostate cancer susceptibility in African-American men: results from the Flint Men’s Health Study. J Hum Genet.

[39] Kibel AS, Jin CH, Klim A, Luly J, A Roehl K, Wu WS, Suarez BK (2008). Association between polymorphisms in cell cycle genes and advanced prostate carcinoma. Prostate.

[40] Hirata H, Kawamoto K, Kikuno N, Kawakami T, Kawakami K, Saini S, Yamamura S, Dahiya R (2009). Bcl2-938C/A polymorphism carries increased risk of biochemical recurrence after radical prostatectomy. J Urol.

[41] Umbas R, Isaacs WB, Bringuier PP, Schaafsma HE, Karthaus HF, Oosterhof GO, Debruyne FM, Schalken JA (1994). Decreased E-cadherin expression is associated with poor prognosis in patients with prostate cancer. Cancer Res.

[42] Dunsmuir WD, Gillett CE, Meyer LC, Young MP, Corbishley C, Eeles RA, Kirby RS (2000). Molecular markers for predicting prostate cancer stage and survival. BJU Int.

[43] Suzuki H, Komiya A, Emi M, Kuramochi H, Shiraishi T, Yatani R, Shimazaki J (1996). Three distinct commonly deleted regions of chromosome arm 16q in human primary and metastatic prostate cancers. Genes Chromosomes Cancer.

[44] Li LC, Chui RM, Sasaki M, Nakajima K, Perinchery G, Au HC, Nojima D, Carroll P, Dahiya R (2000). A single nucleotide polymorphism in the E-cadherin gene promoter alters transcriptional activities. Cancer Res.

[45] Verhage BA, van Houwelingen K, Ruijter TE, Kiemeney LA, Schalken JA (2002). Single-nucleotide polymorphism in the E-cadherin gene promoter modifies the risk of prostate cancer. Int J Cancer.

[46] Arandi N, Talei A, Erfani N, Ghaderi A (2008). Intercellular adhesion molecule-1 genetic markers (+241G/A and +469A/G) in Iranian women with breast cancer. Cancer Genet Cytogenet.

[47] Chen H, Hernandez W, Shriver MD, Ahaghotu CA, Kittles RA (2006). ICAM gene cluster SNPs and prostate cancer risk in African Americans. Hum Genet.

[48] Shirai A, Furukawa M, Yoshizaki T (2003). Expression of intercellular adhesion molecule (ICAM)-1 in adenoid cystic carcinoma of the head and neck. Laryngoscope.

[49] Tachimori A, Yamada N, Sakate Y, Yashiro M, Maeda K, Ohira M, Nishino H, Hirakawa K (2005). Up regulation of ICAM-1 gene expression inhibits tumour growth and liver metastasis in colorectal carcinoma. Eur J Cancer.

[50] Hanahan D, Folkman J (1996). Patterns and emerging mechanisms of the angiogenic switch during tumorigenesis. Cell.

[51] McCarron SL, Edwards S, Evans PR, Gibbs R, Dearnaley DP, Dowe A, Southgate C, Easton DF, Eeles RA, Howell WM (2002). Influence of cytokine gene polymorphisms on the development of prostate cancer. Cancer Res.

[52] Sfar S, Hassen E, Saad H, Mosbah F, Chouchane L (2006). Association of VEGF genetic polymorphisms with prostate carcinoma risk and clinical outcome. Cytokine.

[53] Lee KM, Kang D, Park SK, Berndt SI, Reding D, Chatterjee N, Chanock S, Huang WY, Hayes RB (2009). Nitric oxide synthase gene polymorphisms and prostate cancer risk. Carcinogenesis.

[54] Teixeira AL, Ribeiro R, Cardoso D, Pinto D, Lobo F, Fraga A, Pina F, Calais-da-Silva F, Medeiros R (2008). Genetic polymorphism in EGF is associated with prostate cancer aggressiveness and progression-free interval in androgen blockade-treated patients. Clin Cancer Res.

[55] Tuohimaa P, Tenkanen L, Ahonen M, Lumme S, Jellum E, Hallmans G, Stattin P, Harvei S, Hakulinen T, Luostarinen T, Dillner J, Lehtinen M, Hakama M (2004). Both high and low levels of blood vitamin D are associated with a higher prostate cancer risk: a longitudinal, nested case-control study in the Nordic countries. Int J Cancer.

[56] Ahn J, Albanes D, Peters U, Schatzkin A, Lim U, Freedman M, Chatterjee N, Andriole GL, Leitzmann MF, Hayes RB (2008). RB.Prostate, Lung, Colorectal, and Ovarian Cancer Screening Trial Project Team. Serum vitamin D concentration and prostate cancer risk: a nested case-control study. J Natl Cancer Inst.

[57] Travis RC, Crowe FL, Allen NE, Appleby PN, Roddam AW, Tjønneland A, Olsen A, Linseisen J, Kaaks R, Boeing H, Kröger J, Trichopoulou A, Dilis V, Trichopoulos D, Vineis P, Palli D, Tumino R, Sieri S, Bueno-de-Mesquita HB, van Duijnhoven FJ, Chirlaque MD, Barricarte A, Larrañaga N, González CA, Argüelles MV, Sánchez MJ, Stattin P, Hallmans G, Khaw KT, Bingham S, Rinaldi S, Slimani N, Jenab M, Riboli E, Key TJ (2009). Serum vitamin D and risk of prostate cancer in a case-control analysis nested within the European Prospective Investigation into Cancer and Nutrition (EPIC).. Am J Epidemiol.

[58] Berndt SI, Dodson JL, Huang WY, Nicodemus KK (2006). A systematic review of vitamin D receptor gene polymorphisms and prostate cancer risk. J Urol.

[59] Taylor JA, Hirvonen A, Watson M, Pittman G, Mohler JL, Bell DA (1996). Association of prostate cancer with vitamin D receptor gene polymorphism. Cancer Res.

[60] Ingles SA, Ross RK, Yu MC, Irvine RA, La Pera G, Haile RW, Coetzee GA (1997). Association of prostate cancer risk with genetic polymorphisms in vitamin D receptor and androgen receptor. J Natl Cancer Inst.

[61] Maistro S, Snitcovsky I, Sarkis AS, da Silva IA, Brentani MM (2004). Vitamin D receptor polymorphisms and prostate cancer risk in Brazilian men. Int J Biol Markers.

[62] Cheteri MB, Stanford JL, Friedrichsen DM, Peters MA, Iwasaki L, Lang lois MC, Feng Z, Ostrander EA (2004). Vitamin D receptor gene polymorphisms and prostate cancer risk. Prostate.

[63] Bronner CE, Baker SM, Morrison PT, Warren G, Smith LG, Lescoe MK, Kane M, Earabino C, Lipford J, Lindblom A, Tannergard P, Bollag RJ, Godwin AR, Ward DC, Nordenskj MN, Fishel R, Kolodnerparallel R, Liskay RM (1994). Mutation in the DNA mismatch repair gene homologue hMLH1 is associated with hereditary non-polyposis colon cancer. Nature.

[64] Leach FS, Nicolaides NC, Papadopoulos N, Liu B, Jen J, Parsons R, Peltomaki P, Sistonen P, Aaltonen LA, Nystrom-Lahti M, Guan G, Zhang GI, Meltzer PS, Yu GW, Kao FT, Chen DJ, Cerosaletti KM, Fournier REK, Todd S, Lewis T, Leach RJ, Naylor SL, Weissenbach J, Mecklin JP, Jarvinen H, Petersen GM, Hamilton SR, Green11 J, Jass J, Watson P, Lynch TH, Trent JM, Chapelle ADL, Kinzler KW, Vogelstein B (1993). Mutations of a mutS homolog in hereditary nonpolyposis colorectal cancer. Cell.

[65] Modrich P (1994). Mismatch repair, genetic stability, and cancer. Science.

[66] Aebi S, Kurdi-Haidar B, Gordon R, Cenni B, Zheng H, Fink D, Christen RD, Boland CR, Koi M, Fishel R, Howell SB (1996). Loss of DNA mismatch repair in acquired resistance to cisplatin. Cancer Res.

[67] Cooper CS (2002). Translocations in Solid Tumors. 1st ed.

[68] Tomlins SA, Rhodes DR, Perner S, Dhanasekaran SM, Mehra R, Sun XW, Varambally S, Cao X, Tchinda J, Kuefer R, Lee C, Montie JE, Shah RB, Pienta KJ, Rubin MA, Chinnaiyan AM (2005). Recurrent fusion of TMPRSS2 and ETS transcription factor genes in prostate cancer. Science.

[69] Mosquera JM, Perner S, Demichelis F, Kim R, Hofer MD, Mertz KD, Paris PL, Simko J, Collin C, Bismar TA, Chinnaiyan AM, Rubin MA (2007). Morphological features of TMPRSS2-ERG gene fusion prostate cancer. J Pathol.

[70] Iljin K, Wolf M, Edgren H, Gupta S, Kilpinen S, Skotheim RI, Peltola M, Smit F, Verhaegh G, Schalken J, Nees M, Kallioniemi O (2006). TMPRSS2 fusions with oncogenic ETS factors in prostate cancer involve unbalanced genomic rearrangements and are associated with HDAC1 and epigenetic reprogramming. Cancer Res.

[71] Nam RK, Sugar L, Yang W, Srivastava S, Klotz LH, Yang LY, Stanimirovic A, Encioiu E, Neill M, Loblaw DA, Trachtenberg J, Narod SA, Seth A (2007). Expression of the TMPRSS2:ERG fusion gene predicts cancer recurrence after surgery for localised prostate cancer. Br J Cancer.

[72] Demichelis F, Fall K, Perner S, Andrén O, Schmidt F, Setlur SR, Hoshida Y, Mosquera JM, Pawitan Y, Lee C, Adami HO, Mucci LA, Kantoff PW, Andersson SO, Chinnaiyan AM, Johansson JE, Rubin MA (2007). TMPRSS2: ERG gene fusion associated with lethal prostate cancer in a watchful waiting cohort. Oncogene.

[73] Iljin K, Wolf M, Edgren H, Gupta S, Kilpinen S, Skotheim RI, Peltola M, Smit F, Verhaegh G, Schalken J, Nees M, Kallioniemi O (2006). TMPrss2 fusions with oncogenic eTs factors in prostate cancer involve unbalanced genomic rearrangements and are associated with HDAC1 and epigenetic reprogramming. Cancer Res.

[74] Björkman M, Iljin K, Halonen P, Sara H, Kaivanto E, Nees M, Kallioniemi OP (2008). Defining the molecular action of HDAC inhibitors and synergism with androgen deprivation in erG-positive prostate cancer. Int J Cancer.

[75] Baylin SB, Makos M, Wu JJ, Yen RW, de Bustros A, Vertino P, Nelkin BD (1991). Abnormal patterns of DNA methylation in human neoplasia: potential consequences for tumor progression. Cancer Cells.

[76] Jenuwein T, Allis CD (2001). Translating the histone code. Science.

[77] Marks PA, Rifkind RA, Richon VM, Breslow R (2001). Inhibitors of histone deacetylase are potentially effective anticancer agents. Clin Cancer Res.

[78] Xu W, Cho H, Evans RM (2003). Acetylation and methylation in nuclear receptor gene activation. Methods Enzymol.

[79] Bastian PJ, Ellinger J, Wellmann A, Wernert N, Heukamp LC, Müller SC, von Ruecker A (2005). Diagnostic and prognostic information in prostate cancer with the help of a small set of hypermethylated gene loci. Clin Cancer Res.

[80] Sasaki M, Tanaka Y, Perinchery G, Dharia A, Kotcherguina I, Fujimoto S, Dahiya R (2002). Methylation and inactivation of estrogen, progesterone, and androgen receptors in prostate cancer. J Natl Cancer Inst.

[81] Singal R, Ferdinand L, Reis IM, Schlesselman JJ (2004). Methylation of multiple genes in prostate cancer and the relationship with clinicopathological features of disease. Oncol Rep.

[82] McKie AB, Douglas DA, Olijslagers S, Graham J, Omar MM, Heer R, Gnanapragasam VJ, Robson CN, Leung HY (2005). Epigenetic inactivation of the human sprouty2 (hSPRY2) homologue in prostate cancer. Oncogene.

[83] Jarrard DF, Kinoshita H, Shi Y, Sandefur C, Hoff D, Meisner LF, Chang C, Herman JG, Isaacs WB, Nassif N (1998). Methylation of the androgen receptor promoter CpG island is associated with loss of androgen receptor expression in prostate cancer cells. Cancer Res.

[84] Suzuki H, Ito H (1999). Role of androgen receptor in prostate cancer. Asian J Androl.

[85] Chlenski A, Nakashiro K, Ketels KV, Korovaitseva GI, Oyasu R (2001). Androgen receptor expression in androgen-independent prostate cancer cell lines. Prostate.

[86] Izbicka E, MacDonald JR, Davidson K, Lawrence RA, Gomez L, Von Hoff DD (1999). 5,6 Dihydro-5-azacytidine (DHAC) restores androgen responsiveness in androgen-insensitive prostate cancer cells. Anticancer Res.

[87] Bastian PJ, Yegnasubramanian S, Palapattu GS, Rogers CG, Lin X, De Marzo AM, Nelson WG (2004). Molecular biomarker in prostate cancer: the role ofCpGisland hypermethylation. Eur Urol.

[88] Halkidou K, Gnanapragasam VJ, Meht PB, Logan IR, Brady ME, Cook S, Leung HY, Neal DE, Robson CN (2003). Expression of Tip60, an androgen receptor coactivator, and its role in prostate cancer development. Oncogene.

[89] Debes JD, Schmidt LJ, Huang H, Tindall DJ (2002). p300 mediates androgen-independent transactivation of the androgen receptor by interleukin 6. Cancer Res.

[90] Patra SK, Patra A, Dahiya R (2001). Histone deacetylase and DNA methyltransferase in human prostate cancer. Biochem Biophys Res Commun.

[91] Rosenbaum E, Hoque MO, Cohen Y, Zahurak M, Eisenberger MA, Epstein JI, Partin AW, Sidransky D (2005). Promoter hypermethylation as an independent prognostic factor for relapse in patients with prostate cancer following radical prostatectomy. Clin Cancer Res.

[92] Halkidou K, Gaughan L, Cook S, Leung HY, Neal DE, Robson CN (2004). Upregulation and nuclear recruitment of HDAC1 in hormone refractory prostate cancer. Prostate.

[93] Kirmizis A, Bartley SM, Kuzmichev A, Margueron R, Reinberg D, Green R, Farnham PJ (2004). Silencing of human polycomb target genes is associated with methylation of histone H3 Lys 27. Genes Dev.

[94] Varambally S, Dhanasekaran SM, Zhou M, Barrette TR, Kumar-Sinha C, Sanda MG, Ghosh D, Pienta KJ, Sewalt RG, Otte AP, Rubin MA, Chinnaiyan AM (2002). The polycomb group protein EZH2 is involved in progression of prostate cancer. Nature.

[95] Jarrard DF, Bussemakers MJ, Bova GS, Isaacs WB (1995). Regional loss of imprinting of the insulin-like growth factor II gene occurs in human prostate tissues. Clin Cancer Res.

[96] Fu VX, Schwarze SR, Kenowski ML, Leblanc S, Svaren J, Jarrard DF (2004). A loss of insulin-like growth factor-2 imprinting is modulated by CCCTC-binding factor down-regulation at senescence in human epithelial cells. J Biol Chem.

[97] Lodygin D, Epanchintsev A, Menssen A, Diebold J, Hermeking H (2005). Functional epigenomics identifies genes frequently silenced in prostate cancer. Cancer Res.

[98] Lee WH, Morton RA, Epstein JI, Brooks JD, Campbell PA, Bova GS, Hsieh WS, Isaacs WB, Nelson WG (1994). Cytidine methylation of regulatory sequences near the pi-class glutathione S-transferase gene accompanies human prostatic carcinogenesis. Proc Natl Acad Sci USA.

[99] Nakayama M, Bennett CJ, Hicks JL, Epstein JI, Platz EA, Nelson WG, De Marzo AM (2003). Hypermethylation of the human glutathione S-transferase-pi gene (GSTP1) CpG island is present in a subset of proliferative inflammatory atrophy lesions but not in normal or hyperplastic epithelium of the prostate: a detailed study using laser-capture microdissection. Am J Pathol.

[100] Hoque MO, Topaloglu O, Begum S, Henrique R, Rosenbaum E, Van Criekinge W, Westra WH, Sidransky D (2005). Quantitative methylation-specific polymerase chain reaction gene patterns in urine sediment distinguish prostate cancer patients from control subjects. J Clin Oncol.

[101] Li LC, Okino ST, Dahiya R (2004). DNA methylation in prostate cancer. Biochim Biophys Acta.

[102] Das PM, Singal R (2004). DNA methylation and cancer. J Clin Oncol.

[103] Bastian PJ, Ellinger J, Wellmann A, Wernert N, Heukamp LC, Müller SC, von Ruecker A (2005). Diagnostic and prognostic information in prostate cancer with the help of a small set of hypermethylated gene loci. Clin Cancer Res.

[104] Seligson DB, Horvath S, Shi T, Yu H, Tze S, Grunstein M, Kurdistani SK (2005). Global histone modification patterns predict risk of prostate cancer recurrence. Nature.

[105] Rhodes DR, Sanda MG, Otte AP, Chinnaiyan AM, Rubin MA (2003). Multiplex biomarker approach for determining risk of prostate-specific antigen-defined recurrence of prostate cancer. J Natl Cancer Inst.

[106] Bhalla KN (2005). Epigenetic and chromatin modifiers as targeted therapy of hematologic malignancies. J Clin Oncol.

[107] Goffin J, Eisenhauer E (2002). DNA methyltransferase inhibitors-state of the art. Ann Oncol.

[108] Thibault A, Figg WD, Bergan RC, Lush RM, Myers CE, Tompkins A, Reed E, Samid D (1998). A phase II study of 5-aza-2_deoxycytidine (decitabine) in hormone independent metastatic (D2) prostate cancer. Tumori.

[109] Gudmundsson J, Sulem P, Steinthorsdottir V, Bergthorsson JT, Thorleifsson G, Manolescu A, Rafnar T, Gudbjartsson D, Agnarsson BA, Baker A, Sigurdsson A, Benediktsdottir KR, Jakobsdottir M, Blondal T, Stacey SN, Helgason A, Gunnarsdottir S, Olafsdottir A, Kristinsson KT, Birgisdottir B, Ghosh S, Thorlacius S, Magnusdottir D, Stefansdottir G, Kristjansson K, Bagger Y, Wilensky RL, Reilly MP, Morris AD, Kimber CH, Adeyemo A, Chen Y, Zhou J, So WY, Tong PC, Ng MC, Hansen T, Andersen G, Borch-Johnsen K, Jorgensen T, Tres A, Fuertes F, Ruiz-Echarri M, Asin L, Saez B, van Boven E, Klaver S, Swinkels DW, Aben KK, Graif T, Cashy J, Suarez BK, van Vierssen Trip O, Frigge ML, Ober C, Hofker MH, Wijmenga C, Christiansen C, Rader DJ, Palmer CN, Rotimi C, Chan JC, Pedersen O, Sigurdsson G, Benediktsson R, Jonsson E, Einarsson GV, Mayordomo JI, Catalona WJ, Kiemeney LA, Barkardottir RB, Gulcher JR, Thorsteinsdottir U, Kong A, Stefansson K (2007). Two variants on chromosome 17 confer prostate cancer risk, and the one in TCF2 protects against type 2 diabetes. Nat Genet.

[110] Davis AJ, Gelmon KA, Siu LL, Moore MJ, Britten CD, Mistry N, Klamut H, D'Aloisio S, MacLean M, Wainman N, Ayers D, Firby P, Besterman JM, Reid GK, Eisenhauer EA (2003). Phase I and pharmacologic study of the human DNA methyltransferase antisense oligodeoxynucleotide MG98 given as a 21-day continuous infusion every 4 weeks. Invest New Drugs.

[111] Hu JF, Nguyen PH, Pham NV, Vu TH, and Hoffman AR (1997). Modulation of Igf2 genomic imprinting in mice induced by 5-azacytidine, an inhibitor of DNA methylation. Mol Endocrinol.

[112] Carducci MA, Gilbert J, Bowling MK, Noe D, Eisenberger MA, Sinibaldi V, Zabelina Y, Chen TL, Grochow LB, Donehower RC (2001). A phase I clinical and pharmacological evaluation of sodium phenylbutyrate on an 120-h infusion schedule. Clin Cancer Res.

[113] Kelly WK, O'Connor OA, Krug LM, Chiao JH, Heaney M, Curley T, MacGregore-Cortelli B, Tong W, Secrist JP, Schwartz L, Richardson S, Chu E, Olgac S, Marks PA, Scher H, Richon VM (2005). Phase I study of an oral histone deacetylase inhibitor, suberoylanilide hydroxamic acid, in patients with advanced cancer. J Clin Oncol.

[114] Wang XF, Qian DZ, Ren M, Kato Y, Wei Y, Zhang L, Fansler Z, Clark D, Nakanishi O, Pili R (2005). Epigenetic modulation of retinoic acid receptor beta2 by the histone deacetylase inhibitor MS-275 in human renal cell carcinoma. Clin Cancer Res.

[115] Cheng JC, Yoo CB, Weisenberger DJ, Chuang J, Wozniak C, Liang G, Marquez VE, Greer S, Orntoft TF, Thykjaer T, Jones PA (2004). Preferential response of cancer cells to zebularine. Cancer Cell.

[116] Goffin J, Eisenhauer E (2002). DNA methyltransferase inhibitors-state of the art. Ann Oncol.

[117] Butler LM, Agus DB, Scher HI, Higgins B, Rose A, Cordon-Cardo C, Thaler HT, Rifkind RA, Marks PA, Richon VM (2000). Suberoylanilide hydroxamic acid, an inhibitor of histone deacetylase, suppresses the growth of prostate cancer cells in vitro and in vivo. Cancer Res.

[118] Carducci MA, Nelson JB, Chan-Tack KM, Ayyagari SR, Sweatt WH, Campbell PA, Nelson WG, Simons JW (1996). Phenylbutyrate induces apoptosis in human prostate cancer and is more potent than phenylacetate. Clin Cancer Res.

[119] Mikol YB, Hoover KL, Creasia D, Poirier LA (1983). Hepatocarcinogenesis in rats fed methyl-deficient, amino acid-defined diets. Carcinogenesis.

[120] Harnack L, Jacobs DR Jr, Nicodemus K, Lazovich D, Anderson K, Folsom AR (2002). Relationship of folate, vitamin B-6, vitamin B-12, and methionine intake to incidence of colorectal cancers. Nutr Cancer.

[121] Lyn-Cook BD, Rogers T, Yan Y, Blann EB, Kadlubar FF, Hammons GJ (1999). Chemopreventive effects of tea extracts and various components on human pancreatic and prostate tumor cells in vitro. Nutr Cancer.

[122] Davis CD, Uthus EO, Finley JW (2000). Dietary selenium and arsenic affect DNA methylation in vitro in Caco-2 cells and in vivo in rat liver and colon. J Nutr.

[123] Bedford MT, van Helden PD (1987). Hypomethylation of DNA in pathological conditions of the human prostate.. Cancer Res.

[124] Hanson JA, Gillespie JW, Grover A, Tangrea MA, Chuaqui RF, Emmert-Buck MR, Tangrea JA, Libutti SK, Linehan WM, Woodson KG (2006). Gene promoter methylation in prostate tumor-associated stromal cells. J Natl Cancer Inst.

[125] Henrique R, Jerónimo C, Hoque MO, Carvalho AL, Oliveira J, Teixeira MR, Lopes C, Sidransky D (2005). Frequent 14-3-3 sigma promoter methylation in benign and malignant prostate lesions. DNA Cell Biol.

[126] Witte JS (2007). Multiple prostate cancer risk variants on 8q24. Nat Genet.

[127] Gudmundsson J, Sulem P, Manolescu A, Amundadottir LT, Gudbjartsson D, Helgason A, Rafnar T, Bergthorsson JT, Agnarsson BA, Baker A, Sigurdsson A, Benediktsdottir KR, Jakobsdottir M, Xu J, Blondal T, Kostic J, Sun J, Ghosh S, Stacey SN, Mouy M, Saemundsdottir J, Backman VM, Kristjansson K, Tres A, Partin AW, Albers-Akkers MT, Godino-Ivan Marcos J, Walsh PC, Swinkels DW, Navarrete S, Isaacs SD, Aben KK, Graif T, Cashy J, Ruiz-Echarri M, Wiley KE, Suarez BK, Witjes JA, Frigge M, Ober C, Jonsson E, Einarsson GV, Mayordomo JI, Kiemeney LA, Isaacs WB, Catalona WJ, Barkardottir RB, Gulcher JR, Thorsteinsdottir U, Kong A, Stefansson K (2007). Genome-wide association study identifies a second prostate cancer susceptibility variant at 8q24. Nat Genet.

[128] Haiman CA, Patterson N, Freedman ML, Myers SR, Pike MC, Waliszewska A, Neubauer J, Tandon A, Schirmer C, McDonald GJ, Greenway SC, Stram DO, Le Marchand L, Kolonel LN, Frasco M, Wong D, Pooler LC, Ardlie K, Oakley-Girvan I, Whittemore AS, Cooney KA, John EM, Ingles SA, Altshuler D, Henderson BE, Reich D (2007). Multiple regions within 8q24 independently affect risk for prostate cancer. Nat Genet.

[129] Gudmundsson J, Sulem P, Steinthorsdottir V, Bergthorsson JT, Thorleifsson G, Manolescu A, Rafnar T, Gudbjartsson D, Agnarsson BA, Baker A, Sigurdsson A, Benediktsdottir KR, Jakobsdottir M, Blondal T, Stacey SN, Helgason A, Gunnarsdottir S, Olafsdottir A, Kristinsson KT, Birgisdottir B, Ghosh S, Thorlacius S, Magnusdottir D, Stefansdottir G, Kristjansson K, Bagger Y, Wilensky RL, Reilly MP, Morris AD, Kimber CH, Adeyemo A, Chen Y, Zhou J, So WY, Tong PC, Ng MC, Hansen T, Andersen G, Borch-Johnsen K, Jorgensen T, Tres A, Fuertes F, Ruiz-Echarri M, Asin L, Saez B, van Boven E, Klaver S, Swinkels DW, Aben KK, Graif T, Cashy J, Suarez BK, van Vierssen Trip O, Frigge ML, Ober C, Hofker MH, Wijmenga C, Christiansen C, Rader DJ, Palmer CN, Rotimi C, Chan JC, Pedersen O, Sigurdsson G, Benediktsson R, Jonsson E, Einarsson GV, Mayordomo JI, Catalona WJ, Kiemeney LA, Barkardottir RB, Gulcher JR, Thorsteinsdottir U, Kong A, Stefansson K (2007). Two variants on chromosome 17 confer prostate cancer risk, and the one in TCF2 protects against type 2 diabetes. Nat Genet.

[130] Duggan D, Zheng SL, Knowlton M, Benitez D, Dimitrov L, Wiklund F, Robbins C, Isaacs SD, Cheng Y, Li G, Sun J, Chang BL, Marovich L, Wiley KE, Bälter K, Stattin P, Adami HO, Gielzak M, Yan G, Sauvageot J, Liu W, Kim JW, Bleecker ER, Meyers DA, Trock BJ, Partin AW, Walsh PC, Isaacs WB, Grönberg H, Xu J, Carpten JD (2007). Two genome-wide association studies of aggressive prostate cancer implicate putative prostate tumor suppressor gene DAB2IP. J Natl Cancer Inst.

